# Socioeconomic and demographic determinants of radiation treatment and outcomes in glioblastoma patients

**DOI:** 10.3389/fneur.2022.1024138

**Published:** 2022-11-11

**Authors:** Eric J. Hsu, Jamie Thomas, Robert D. Timmerman, Zabi Wardak, Tu D. Dan, Toral R. Patel, Nina N. Sanford, Dat T. Vo

**Affiliations:** ^1^Department of Radiation Oncology, UT Southwestern Medical Center, Dallas, TX, United States; ^2^Department of Neurological Surgery, UT Southwestern Medical Center, Dallas, TX, United States

**Keywords:** radiation therapy, glioblastoma, socioeconomic, age, insurance, depressive disorder

## Abstract

**Introduction:**

Poor outcomes in glioblastoma patients, despite advancing treatment paradigms, indicate a need to determine non-physiologic prognostic indicators of patient outcome. The impact of specific socioeconomic and demographic patient factors on outcomes is unclear. We sought to identify socioeconomic and demographic patient characteristics associated with patient survival and tumor progression, and to characterize treatment options and healthcare utilization.

**Methods:**

A cohort of 169 patients with pathologically confirmed glioblastomas treated at our institution was retrospectively reviewed. Multivariable cox proportional hazards analysis for overall survival (OS) and cumulative incidence of progression was performed. Differences in treatment regimen, patient characteristics, and neuro-oncology office use between different age and depressive disorder history patient subgroups were calculated two-sample *t-*tests, Fisher's exact tests, or linear regression analysis.

**Results:**

The median age of all patients at the time of initiation of radiation therapy was 60.5 years. The median OS of the cohort was 13.1 months. Multivariable analysis identified age (Hazard Ratio 1.02, 95% CI 1.00–1.04) and total resection (Hazard Ratio 0.52, 95% CI 0.33–0.82) as significant predictors of OS. Increased number of radiation fractions (Hazard Ratio 0.90, 95% CI 0.82–0.98), depressive disorder history (Hazard Ratio 0.59, 95% CI 0.37–0.95), and total resection (Hazard Ratio 0.52, 95% CI 0.31–0.88) were associated with decreased incidence of progression. Notably, patients with depressive disorder history were observed to have more neuro-oncology physician office visits over time (median 12 vs. 16 visits, *p* = 0.0121). Patients older than 60 years and those with Medicare (vs. private) insurance were less likely to receive as many radiation fractions (*p* = 0.0014) or receive temozolomide concurrently with radiation (Odds Ratio 0.46, *p* = 0.0139).

**Conclusion:**

Older glioblastoma patients were less likely to receive as diverse of a treatment regimen as their younger counterparts, which may be partially driven by insurance type. Patients with depressive disorder history exhibited reduced incidence of progression, which may be due to more frequent health care contact during neuro-oncology physician office visits.

## Introduction

Glioblastomas are the most common primary intracranial tumor, accounting for more than 40% of all malignant brain tumors (1). Glioblastoma affects 3.23 persons per 100,000 in the United States every year (2). Management of glioblastomas entails multimodal treatment involving combinations of surgical resection, adjuvant radiation therapy, and adjuvant chemotherapy, with the treatment paradigm constantly evolving (3). However, despite advancing treatments, glioblastoma patients still have a poor prognosis and exhibit a 5-year survival of only ~5% after initial diagnosis (4, 5). As survival in cancer patients, especially those with glioblastomas, is multifactorial, it becomes vital to consider not only direct treatment efficacy, but also non-physiologic patient factors.

Numerous studies have assessed how socioeconomic or demographic patient characteristics contribute to glioblastoma patient outcome. For example, glioblastoma patients who are married exhibit improved survival outcomes compared to widowed or divorced patients, with this better prognosis being attributed to better social support and wellness (6). Patients who hold private insurance rather than Medicaid or no insurance have more treatment options and exhibit longer survival (7, 8). Moreover, having a primary care physician and receiving subsequent treatment after surgical tumor resection have also been associated with improved patient prognosis (9, 10). Such studies indicate that glioblastoma patients who ultimately have better access to medical care have better prognosis. However, the specific aspects of healthcare availability that are related to such patient characteristics requires further elucidation. How treatment options differ between different demographic and socioeconomic subgroups should be assessed. Furthermore, a direct quantification of how much patients ultimately utilize their healthcare resources (i.e., office visits) should be incorporated. Improved knowledge on these relationships would better inform clinicians on areas of improvement that would more effectively address patient needs and personalize their care. Therefore, this study investigates which socioeconomic and demographic patient characteristics predict for patient survival and incidence of progression. We subsequently evaluate how treatment modalities and healthcare utilization differ within patient subgroups (i.e., age, mental disorder history, and insurance status) and how this correlates to clinical outcomes.

## Methods

A database of 169 patients with primary brain glioblastomas treated with radiation therapy at UT Southwestern between May 2015 and February 2021 was retrospectively reviewed. Patients underwent pathological typing according to the 2016 WHO Classification of Tumors of the Central Nervous System (11). Patient data was obtained through electronic medical record review. The study was approved by the UT Southwestern Institutional Review Board (IRB number STU 062014-027).

All patients received radiation therapy targeted to either the primary tumor or the post-tumor resection cavity, with patients receiving doses ranging from 16 to 75 Gy in 4 to 30 fractions. Patients underwent CT simulation with a tailored head-thermoplastic mask in the supine position. A gross tumor volume (GTV) is delineated using a fused postoperative MRI on the T1 and T2 FLAIR sequences, followed by a creation of a clinical target volume (CTV) to cover the potential areas of microscopic disease. Then, a planning target volume (PTV) expansion was created to account for daily uncertainty in daily set-up and treatment delivery, per our institutional protocol and standards.

We then assessed the patterns of failure, including in-field failures (within the 95% isodose volume), out-of-field failures, or marginal failures (within the 50–95% isodose volume) as observed radiographically on MRI. Incidence of progression was defined as any instance of these failures. Time to progression/recurrence was defined as the time from the end of the radiation treatment period to the first radiographic evidence of recurrence.

### Statistics

Overall survival (OS), progression-free survival (PFS), and cumulative incidence of progression were estimated using Kaplan-Meier method. Patients who were alive without evidence of recurrence were censored at the date of last follow up. *P-*values were calculated from incidence of recurrence or death and survival curves were created with Cox proportional hazards tests. *P-*values were considered significant at <0.05.

Specific measures of socioeconomic status in this study included patient housing status and employment. To quantify housing status, a housing score, based on the HOUSES score, was calculated (12, 13). This housing score was calculated as the sum of *Z* transformations of the square footage, number of bedrooms, number of bathrooms, and appraised property value of each patient's living situation. Appraised property values were obtained *via* publicly accessible county appraisal databases. Patients who rented apartments had their housing score factors estimated based on public apartment floor plan pricing and details.

Cox proportional hazards regression was used to determine the impact of patient covariates on OS and cumulative incidence of progression. Hazard ratios and confidence intervals were calculated for each variable. Multivariable Cox proportional hazards regression models were used to adjust for patient characteristics (age, body mass index, distance from home to clinic, radiation dose, number of radiation fractions, housing score, gender, race, marital status, employment status, insurance status, smoking history, anxiety disorder history, depressive disorder history, total resection status, concurrent temozolomide use during radiation, and concurrent dexamethasone use during radiation).

Patients were also split into subgroups by either age (less than or greater than 60 years) or depressive disorder history prior to cancer diagnosis (positive or negative). For each subgroup, for patient characteristics considered as continuous variables (age, body mass index, distance from home to clinic, radiation dose, number of radiation fractions, or housing score), two sample *t-*tests were used to calculate differences between each patient cohort. For categorical variables (gender, race, histology, marital status, employment status, insurance status, smoking history, anxiety disorder history, depressive disorder history, total resection status, concurrent temozolomide use during radiation, or concurrent dexamethasone use during radiation), Fisher's exact tests were used to calculate the odds ratios between different patient characteristics. Office visits were defined as visits to a physician in a neurology, neurological surgery, or radiation oncology specialty. Linear regression was performed to determine the relationship between total number of office visits and time since initial office visit.

## Results

The median age of all patients at the time of initiation of radiation therapy was 60.5 years (range 23.5–84.3). Other patient demographics, socioeconomic status, and medical history are detailed in Table 1. Patients were treated with 16 to 75 Gy radiation therapy in 4 to 30 fractions, and the most commonly used regimen was 60 Gy in 30 fractions. Median follow-up for all patients was 12.3 months (range = 0.2–66.7 months).

**Table 1:** Patient characteristics.

**Characteristics**	**Cohort**
Total patients	169
**Age (years)**	
Median	60.5
Range	23.5–84.3
**BMI**	
Median	28.2
Range	18.8–48.8
**Home distance from clinic (miles)**	
Median	26.5
Range	1.8–637.0
**Dose (Gy)**	
Median	54.0
Range	16.0–75.0
**Number of fractions**	
Median	30
Range	4–30
**Gender**	
Male	106
Female	63
**Race**	
Caucasian	152
African American	10
Asian	7
**Marriage status**	
Married	133
Not married	36
**Employment status**	
Employed	70
Unemployed	68
Retired	31
**Home ownership status**	
Own	161
Rent	9
**Insurance status**	
Private insurance	106
Medicare	63
**Smoking history**	
Positive	38
Negative	131
**Anxiety disorder history**	
Positive	30
Negative	139
**Depressive disorder history**	
Positive	76
Negative	93
**Total surgical resection status**	
Positive	129
Negative	40
**Concurrent temozolomide use**	
Positive	81
Negative	88
**Concurrent dexamethasone use**	
Positive	76
Negative	93
**Follow up duration (months)**	
Median	12.3
Range	0.2–66.7

Gy, Gray; BMI, Body Mass Index.

**Figure 1:**
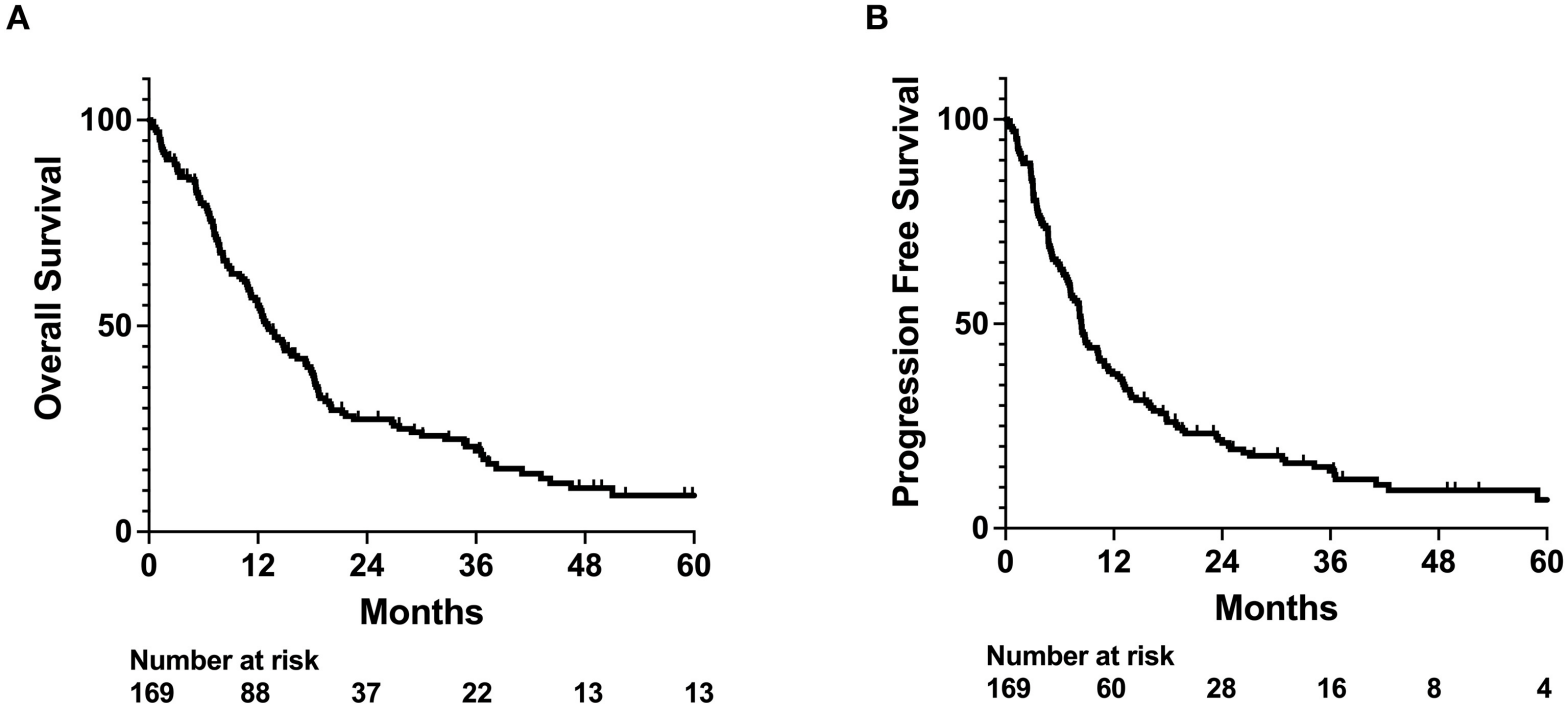
OS and PFS outcomes for whole patient cohort. Kaplan-Meyer plot of **(A)** overall survival (OS) and **(B)** progression free survival (PFS).

For the entire cohort, median OS and PFS since the end of radiation treatment were 13.1 and 8.4 months (Figure 1), respectively. On univariable analysis, older age and Medicare insurance status were associated with worse OS while depressive disorder history was associated with decreased progression (Figure 1; Supplementary Table 1). Increased dose, increased number of fractions, and total resection status were treatment modalities associated with improved survival and decreased incidence of progression (Supplementary Table 1; Supplementary Figure 1). However, after adjustment for other patient characteristics, only age and total resection status predicted for OS on both univariable and multivariable analysis, while number of fractions, depressive disorder history, and total resection status predicted for incidence of progression (Table 2). Increased age was associated with only worse OS (Adj. Hazard Ratio 1.024/year, 95% CI 1.004–1.043, *p* = 0.0172) but not incidence of progression. Increased number of fractions (Adj. Hazard Ratio 0.898/fraction, 95% CI 0.823–0.981, *p* = 0.0169) and depressive disorder history (Adj. Hazard Ratio 0.588, 95% CI 0.366–0.946, *p* = 0.0285) were associated with decreased incidence of progression. Patients who received total resection for their tumors had better OS (Adj. Hazard Ratio 0.516, 95% CI 0.326–0.816, *p* = 0.0047) and reduced time to tumor progression (Adj. Hazard Ratio 0.520, 95% CI 0.307–0.880, *p* = 0.0150).

**Table 2:** Multivariable analysis of impact of patient characteristics on OS and Cumulative Incidence of Progression in the full patient cohort.

**Variable**	**OS**			**Cumulative incidence**		
	**Hazard ratio (Adj.)**	**95% CI**	***p*-value**	**Hazard ratio (Adj.)**	**95% CI**	***p*-value**
Age	1.024	1.004–1.043	0.0172	1.003	0.983–1.024	0.7391
BMI	0.973	0.938–1.009	0.1445	1.006	0.967–1.046	0.7621
Distance from clinic	0.999	0.997–1.002	0.5891	0.998	0.995–1.001	0.2810
Dose (Gy)	0.988	0.944–1.035	0.6162	1.046	0.993–1.102	0.0873
Fractions	0.980	0.910–1.056	0.5966	0.898	0.823–0.981	0.0169
Housing score	1.007	0.958–1.058	0.7861	1.014	0.954–1.078	0.6479
Gender (Male)	1.566	1.054–2.327	0.0265	1.521	0.964–2.402	0.0716
Race (Caucasian)	0.655	0.348–1.230	0.1880	0.875	0.430–1.783	0.7134
Marital status (Married)	1.162	0.717–1.882	0.5431	1.292	0.744–2.244	0.3634
Employment (Employed)	0.872	0.531–1.431	0.5875	0.755	0.427–1.336	0.3345
Insurance (Private)	1.012	0.585–1.751	0.9666	1.336	0.701–2.546	0.3780
Smoking history	0.881	0.546–1.421	0.6036	0.579	0.324–1.035	0.0650
Anxiety disorder	1.400	0.806–2.434	0.2323	1.498	0.791–2.839	0.2148
Depressive disorder	0.820	0.546–1.232	0.3391	0.588	0.366–0.946	0.0285
Total resection	0.516	0.326–0.816	0.0047	0.520	0.307–0.880	0.0150
Temozolomide use	1.028	0.708–1.491	0.8857	0.812	0.525–1.254	0.3475
Dexamethasone use	1.387	0.926–2.078	0.1125	1.201	0.755–1.910	0.4388

Cox Proportional Hazards Regression was performed on multivariable models on the listed variables. Hazard ratios for age, BMI, dose, fractions, distance from home to clinic, and housing score were calculated as continuous variables, while the remaining variables were calculated as categorical variables. OS, Overall Survival; Gy, Gray; BMI, Body Mass Index; CI, Confidence Interval.

As age and depressive disorder history were associated with patient outcome, we performed further investigation to determine why older patients and patients without depressive disorder history potentially had worse outcome. We observed that patients who were older than 60 years old were less likely to be employed (*p* < 0.0001), received fewer radiation treatments (*p* = 0.0014), and were less likely to receive concurrent temozolomide (Odds Ratio 0.4555, *p* = 0.0139) (Table 3; **Figures 3A,B**). Older patients also had fewer neurology, neurological surgery, or radiation oncology office visits (*p* = 0.0089) than their younger counterparts (**Figure 3C**). However, the amount of office visits normalized for total time from initial to final office visit were not significantly different (**Figure 3D**). Patients with Medicare insurance were also significantly associated with older age, and such patients also received fewer radiation treatments and less temozolomide than their private insurance counterparts (Table 3; Supplementary Table 2; Supplementary Figure 2). Furthermore, patients with Medicare insurance were less likely to be non-Caucasian or employed (Supplementary Table 2).

**Table 3:** Patient characteristics in younger vs. older patients.

**Characteristics**	**Age ≤ 60**	**Age > 60**	**p value**
Total patients	81	88	
**BMI**
Median	28.5	27.6	0.7782
Range	18.8–48.8	18.8–45.7	
**Home distance from clinic (miles)**
Median	26.4	26.6	0.3934
Range	1.8–395.0	1.8–637.0	
**Dose (Gy)**
Median	57	45	0.0609
Range	20–75	16–75	
**Number of fractions**
Median	30	23	0.0014
Range	5–30	4–30	
**Housing score**
Median	−0.55	−0.70	0.9230
Range	−5.61–9.02	−5.61–19.60	
**Gender**
Male	51	55	0.9999
Female	30	33	
**Race**
Caucasian	70	82	0.2008
Non-caucasian	11	6	
**Marriage status**
Married	61	72	0.3493
Not married	20	16	
**Employment status**
Employed	54	16	<0.0001
Unemployed	27	72	
**Insurance status**
Private insurance	79	27	<0.0001
Medicare	2	61	
**Smoking history**
Positive	15	23	0.2712
Negative	66	65	
**Anxiety disorder history**
Positive	18	12	0.1624
Negative	63	76	
**Depressive disorder history**
Positive	37	39	0.7201
Negative	44	49	
**Total surgical resection status**
Positive	60	69	0.5878
Negative	21	19	
**Concurrent temozolomide use**
Positive	47	34	0.0139
Negative	34	54	
**Concurrent dexamethasone use**
Positive	34	42	0.5361
Negative	47	46	

Differences between the number of patients for each characteristic between the two cohorts were calculated. BMI, distance from home to clinic, dose, fractions, and housing score were calculated as continuous variables, while the remaining variables were calculated as categorical variables. P-values were calculated with two sample t-tests for continuous variables and Fisher's exact tests for categorical variables. G, Gray; BMI, Body Mass Index.

Radiation, surgery, chemotherapy, and steroid use between patients without or with depressive disorder history did not significantly differ (Table 4; **Figures 4A,B**). Patients who had depression were modestly associated with living further from clinic (*p* = 0.0850), being Caucasian (Odds Ratio 2.925, *p* = 0.0741), and being unmarried (Odds Ratio 0.5037, *p* = 0.0891). Lastly, patients with depressive disorder history had more total office visits (*p* = 0.0121) and total office visits normalized for total time (slope 95% CI 0.4624–0.5953 for no depression vs. 0.6242–0.7680 for depression) than those without depressive disorder history (**Figures 4C,D**).

**Table 4:** Patient characteristics in patients without vs. with history of depressive disorder.

**Characteristics**	**No depression**	**Depression**	**p value**
Total patients	93	76	
**Age (years)**
Median	62.0	60.3	0.6708
Range	24.5–83.4	23.5–84.3	
**BMI**
Median	28.2	28.0	0.1820
Range	19.1–48.8	18.8–42.9	
**Home distance from clinic (miles)**
Median	25.7	27.8	0.0850
Range	1.8–637.0	1.8–395.0	
**Dose (Gy)**
Median	46.0	49.0	0.4594
Range	16.0–75.0	20.0–75.0	
**Number of fractions**
Median	23	25	0.4043
Range	4–30	5–30	
**Housing score**
Median	−0.00	−1.44	0.2500
Range	−5.61 to 19.60	−5.61 to 8.74	
**Gender**
Male	58	48	0.9999
Female	35	28	
**Race**
Caucasian	80	72	0.0741
Non-caucasian	13	4	
**Marriage status**
Married	78	55	0.0891
Not married	15	21	
Employment status
Employed	51	48	0.3464
Unemployed	42	28	
**Insurance status**
Private insurance	57	49	0.7497
Medicare	36	27	
**Smoking history**
Positive	19	19	0.5790
Negative	74	57	
**Anxiety disorder history**
Positive	6	24	<0.0001
Negative	87	52	
**Total surgical resection status**
Positive	68	61	0.3632
Negative	25	15	
**Concurrent temozolomide use**
Positive	45	36	0.9999
Negative	48	40	
**Concurrent dexamethasone use**
Positive	41	35	0.8767
Negative	52	41	

Differences between the number of patients for each characteristic between the two cohorts were calculated. Age, BMI, distance from home to clinic, dose, fractions, and housing score were calculated as continuous variables, while the remaining variables were calculated as categorical variables. P-values were calculated with two sample t-tests for continuous variables and Fisher's exact tests for categorical variables. Gy, Gray; BMI, Body Mass Index.

## Discussion

Glioblastomas are the most prevalent adult brain tumor, and despite advances in therapy, glioblastomas are nearly universally fatal. Therefore, it is vital to determine not only which factors, including socioeconomic status, accurately contribute to poor outcome, but also how such factors contribute to patient survival. With how surgery and adjuvant treatments can increase survival in glioblastoma patients, it becomes vital to understand how non-physiologic factors affect treatment selection (14). Our study thus evaluates the interplay between socioeconomic and demographic factors, treatment regimen, and patient outcome.

**Figure 2:**
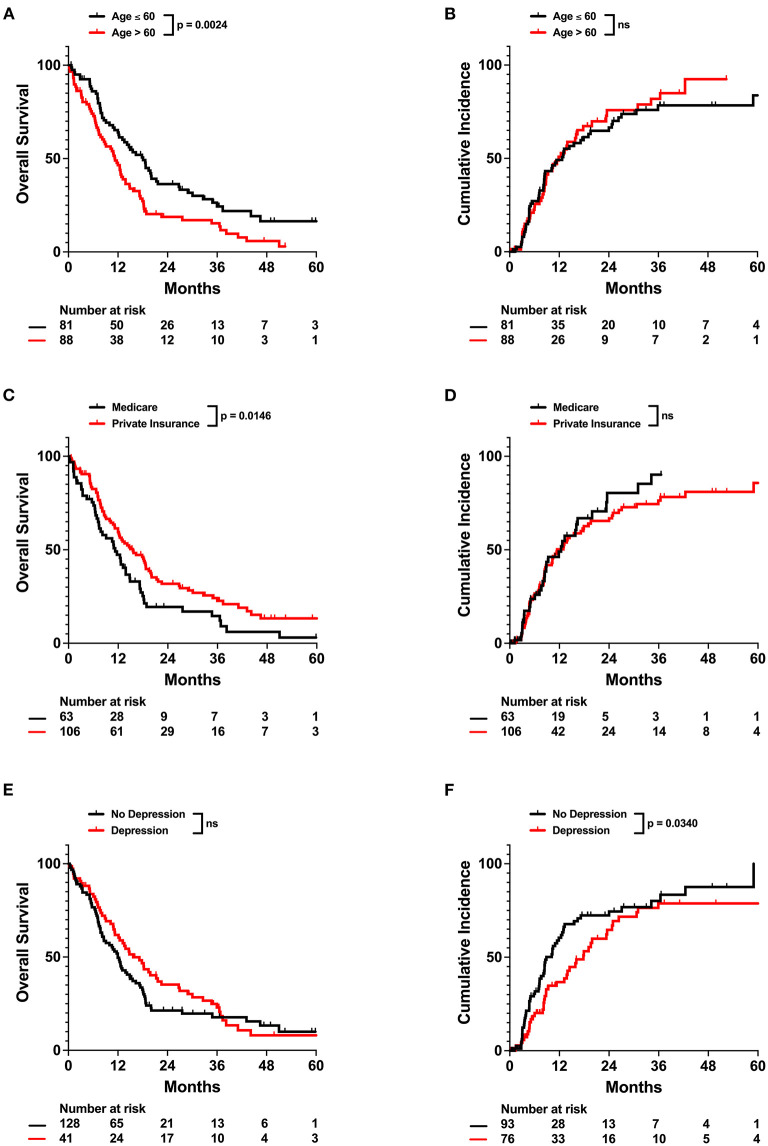
Univariate impact of age, insurance status, and depressive disorder on OS and Cumulative Incidence in the full patient cohort. Kaplan-Meyer plots of OS and Cumulative Incidence comparing patients with different **(A,B)** age, **(C,D)** insurance status, or **(E,F)** depressive disorder history. Statistical analysis was performed using Cox Proportional Hazards tests. OS, overall survival, NS, not significant.

**Figure 3:**
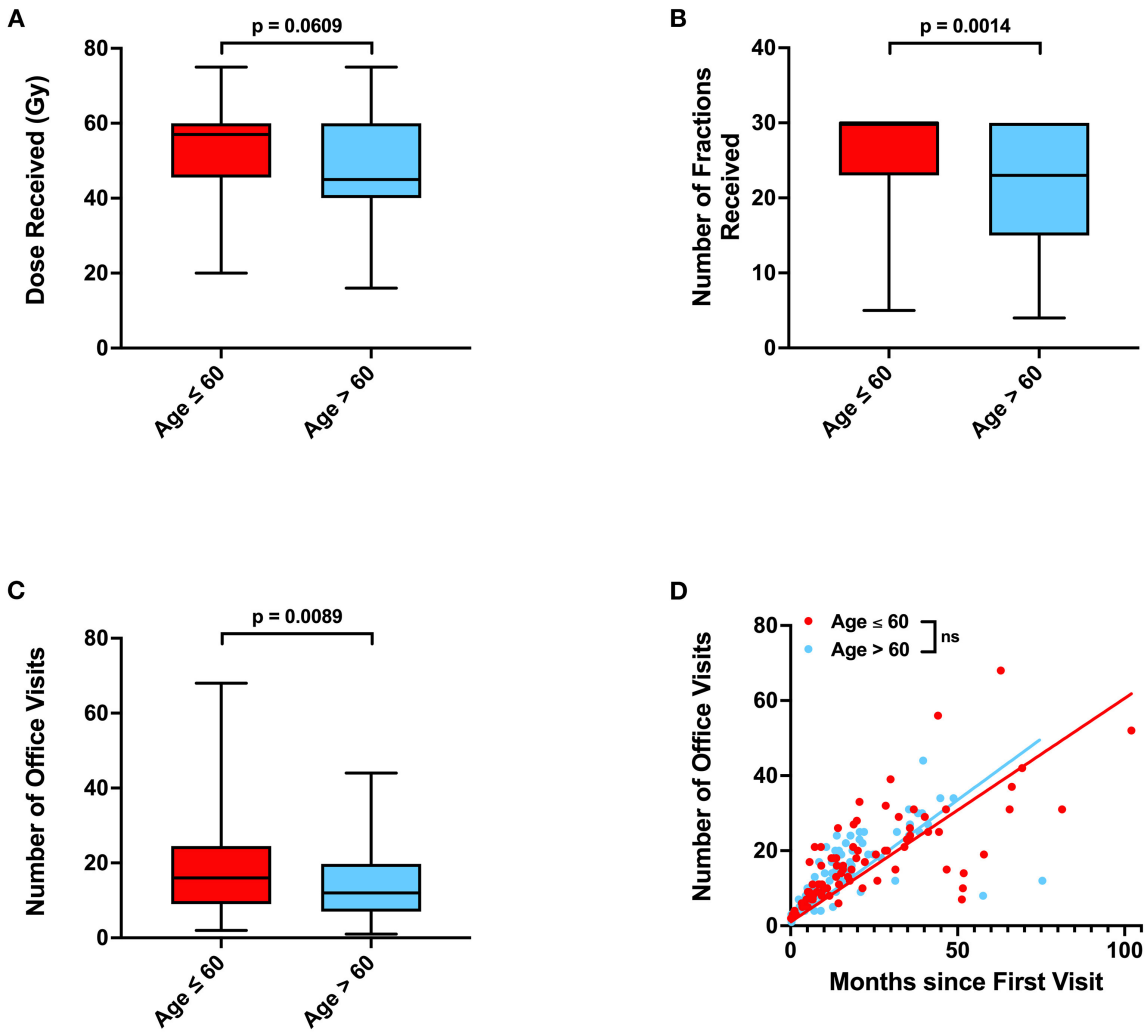
Associations between age, radiation treatments, and office visits. **(A)** Radiation dose, **(B)** number of radiation fractions, and **(C)** total number of neurology, neurological surgery, or radiation oncology office visits in patients of age ≤ 60 or >60. **(D)** Relationship between total time elapsed from first to final office visits and total number of office visits for each patient, with calculated linear regression line and *P-*values for slope differences. 95% CI for linear regression slope is 0.5270–0.6647 for patients age ≤ 60 vs. 0.5738–0.7270 for patients age >60. *P-*values for were calculated with two sample *t*-tests. Gy, Gray, NS, not significant.

Our multivariable analysis of non-physiologic predictors of survival in our full patient cohort yielded younger age and total tumor resection as significant predictors of improved survival, which is consistent with previous studies (15). Increased number of radiation fractions and history of depressive disorder contributed to improved progression outcomes (Figure 2; Table 2). As radiation fractions and surgery are standard of care glioblastoma treatment modalities that have previously been demonstrated to improve patient outcomes, we turned toward better understanding how patient populations of different age and depressive disorder history varied (15, 16). We observed that patients older than 60 years old received fewer radiation fractions than younger patients (Table 3; Figure 3). Furthermore, fewer older patients received concurrent temozolomide therapy with radiation (Table 3). These results suggest that older patients are less likely to receive more intense treatment regimens than younger patients. The reason behind this may be multifactorial. Patients who are older may favor shorter, convenient treatment regimens than standard radiation treatment courses for a multitude of reasons. Potential elderly age-related medical comorbidities, impaired performance status, discomfort during treatment setup, or lack of continued income after retirement to pay for medical treatments may discourage them from receiving long courses of radiation. Previous studies have demonstrated that such hypofractionated and dose de-escalated regimens are noninferior to conventional radiation treatment courses in elderly patients in terms of patient survival (17–19). While our study similarly observed no significant relationship between radiation treatment regimen and survival, we did observe an increase in tumor progression in patients receiving less radiation (Figure 2; Table 2). In addition, the use of concurrent temozolomide was only recently demonstrated to have a survival benefit, as it was unknown if the addition of temozolomide was beneficial for older patients receiving shorter course of radiation therapy (20). Older patients, especially those over age 65, may also have less access to more diverse healthcare options, which may be due to poorer insurance coverage. Very interestingly, in our study, we observed that glioblastoma patients with Medicare similarly received less radiation and chemotherapy treatment than those who had private insurance, which is consistent with previous studies (Supplementary Table 2; Supplementary Figure 2) (7). While we do note this significant difference, the overall lack of significant survival benefit in patients with private insurance may be in part due to a lack of uninsured or Medicaid insured patients in our patient cohort. These patients may lack treatment accessibility or options even further than those who are Medicare insured, which could ultimately result in poorer prognosis (7).

**Figure 4:**
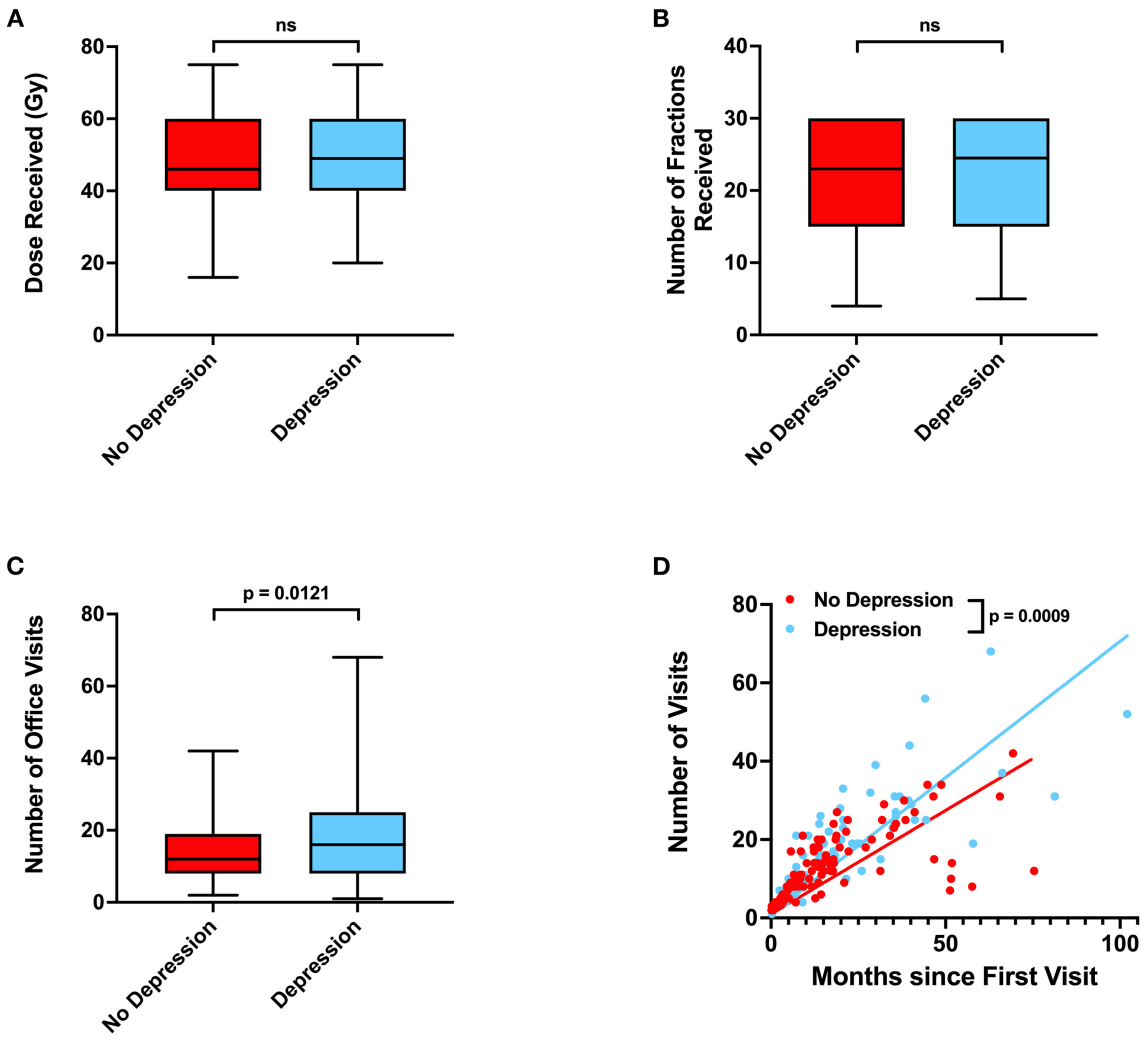
Associations between depressive disorder history, radiation treatments, and office visits. **(A)** Radiation dose, **(B)** number of radiation fractions, and **(C)** total number of neurology, neurological surgery, or radiation oncology office visits in patients without or with depressive disorder history. **(D)** Relationship between total time elapsed from first to final office visits and total number of office visits for each patient, with calculated linear regression line and *P-*values for slope differences. 95% CI for linear regression slope is 0.4624–0.5953 for patients without depression vs. 0.6242–0.7680 for patients with depression. *P-*values for were calculated with two sample *t*-tests. Gy, Gray, NS, not significant.

Unexpectedly, we also observed that depressive disorder history was associated with decreased incidence of progression in our patient cohort (Table 2). Patients who have glioblastoma may present with mood changes, such as depression, prior to diagnosis or due to receipt of news of a cancer diagnosis (21, 22). Depression has been previously associated with poor prognosis in glioblastoma patients (23). Our results suggest that patients with history of depression do exhibit some trends, such as being unmarried, that suggest difficulty receiving social support (Table 4) (24). However, we also observe that patients with depression have a statistically significantly greater number of neurology, neurological surgery, or radiation oncology office visits and more frequent office visits over time (Figures 4C,D). These results suggest that glioblastoma patients with depression ultimately have greater physician office visit utilization in specialties related to their CNS cancers despite potentially having more limited mental and social support. Such frequent visits may be due to more consistent monitoring of depression as a less imminently lethal comorbidity of glioblastoma, thus resulting in concurrent surveillance of cancer progression. Previous studies have also observed that cancer patients with depression have greater healthcare utilization, which may potentially lead to more prompt care of oncologic disease complications (25).

Indicators of socioeconomic status that have previously been identified include housing status, occupation and employment, education level, and income (26). These indicators have been included in studies that evaluate the relationship between socioeconomic status and overall cancer survival. Income level has been observed in previous studies to be associated with poor patient survival in the general cancer and glioblastoma patient populations (27–29). Low housing value, at least in the Singaporean population, has also been observed to be correlated with poor breast cancer patient survival (30). From our patient database, we assessed the housing and employment status of each patient as indicators of socioeconomic status. Our housing score was based off the HOUSES score, which has previously suggested to be a good surrogate measure of socioeconomic status (12, 13). However, in our study, we did not observe housing score or employment status to significantly predict for overall survival or incidence of progression. This in part may be due to the lack of patients who had particularly poor socioeconomic status, as many of our patients were homeowners or all of our patients had sufficient resources to maintain a place to live. On the other hand, it is difficult to determine the true socioeconomic and even housing status of our patients. Cancer, especially GBM as it is a relatively high morbidity cancer, can potentially become debilitating enough to cause patients to stop working. The impacts of unemployment and the high costs of cancer treatment mentally and financially not only affect quality of life but may also influence patients who may not necessarily be of high socioeconomic status to live with the family members who are most well off and capable of helping them achieve proper healthcare.

Our study had the traditional limitations that are intrinsic to all retrospective studies. These limitations include non-random treatment group allocation, selection bias, and non-random loss to follow up, all of which are present in any non-randomized non-prospective study (31). While our study factors in loss to follow up during statistical analysis, the patient cohort is biased toward being more affluent which is evidenced by the capability of all patients being able to receive radiation and the lack of homeless and uninsured or Medicaid insured patients. Furthermore, as age and insurance were so strongly correlated, we could not effectively distinguish which of these two factors more definitively affected both first- and second-line treatment regimens. Moreover, these patient characteristics may also potentially be associated with aggressive tumor biology, extent of disease, or poor performance status. Such tumor biomarkers and prognostic factors were not assessed in this study. Income and education level were also patient socioeconomic characteristics that were not as readily available and thus not included here. Lastly, our patient sample size was relatively small, especially for a socioeconomic evaluation. Our patient population was mostly Caucasian and thus lacked racial diversity, which especially given the lack of non-Caucasian patients due to a smaller patient sample size may limit the generalization of our conclusions to more diverse populations of patients.

Overall, in our full patient cohort, older age and lack of total tumor resection independently predicted for worse survival while fewer radiation fractions and absence of depressive disorder predicted for greater incidence of progression. Older patients received fewer radiation treatments and less chemotherapy use than their younger counterparts. History of depressive disorder provided a protective prognostic effect for glioblastoma patients, which can potentially be attributed to increased healthcare use and neuro-oncology physician communication. Taken together, our study assesses the socioeconomic and demographic factors that affect access to sufficient treatment, utilization of healthcare, and outcome in glioblastoma patients. Such investigation can inform clinicians on how to address issues associated with limited glioblastoma patient access to care especially as treatment paradigms continue to advance.

## Data availability statement

The raw data supporting the conclusions of this article will be made available by the authors, without undue reservation.

## Ethics statement

The studies involving human participants were reviewed and approved by UT Southwestern Institutional Review Board. Written informed consent for participation was not required for this study in accordance with the national legislation and the institutional requirements.

## Author contributions

Conceptualization: EH, NS, and DV. Methodology: EH, JT, and DV. Formal analysis: EH. Investigation and draft review: EH, JT, RT, ZW, TP, TD, NS, and DV. Original draft: EH and DV. Supervision: DV. All authors contributed to the article and approved the submitted version.

## Funding

The study data were collected and managed using the Clinical Data Exchange Network (ClinDEN) hosted by UT Southwestern Medical Center and supported by CTSA Grant Number UL1 TR003163 from the National Center for Advancing Translational Science (NCATS) and a component of the National Institutes of Health (NIH).

## Conflict of interest

Author RT is on the board of directors for TRIO Corporation, TMIT Corporation, and Reflexion. Author DV has research funding from AstraZeneca. All do not relate to the subject of this study. The remaining authors declare that the research was conducted in the absence of any commercial or financial relationships that could be construed as a potential conflict of interest.

## Publisher's note

All claims expressed in this article are solely those of the authors and do not necessarily represent those of their affiliated organizations, or those of the publisher, the editors and the reviewers. Any product that may be evaluated in this article, or claim that may be made by its manufacturer, is not guaranteed or endorsed by the publisher.

## Author disclaimer

The content is solely the responsibility of the authors and does not necessarily represent the official views of the NIH.
