# Supplementary material for: Socioeconomic and demographic determinants of radiation treatment and outcomes in glioblastoma patients
Source: Front Neurol. 2022 Nov 11;13:1024138. doi: 10.3389/fneur.2022.1024138 (PMC9691959; doi:10.3389/fneur.2022.1024138)
Supplement: Supplementary file 1 [file Table_1.DOCX]

**Supplementary Data**

**Supplementary Table 1.** Univariate analysis of impact of patient characteristics on OS and Cumulative Incidence in the full patient cohort. Cox Proportional Hazards Regression was performed on univariate models on the listed variables. Hazard ratios for age, BMI, dose, fractions, distance from home to clinic, and housing score were calculated as continuous variables, while the remaining variables were calculated as categorical variables. Abbreviations: OS = Overall Survival, Gy = Gray, BMI = Body Mass Index, CI = Confidence Interval.

**Supplementary Figure 1.** Univariate impact of radiation dose, number of fractions, and total resection status on OS and Cumulative Incidence in the full patient cohort. Kaplan-Meyer plots of OS and Cumulative Incidence comparing patients with different **(A-B)** radiation dose, **(C-D)** number of fractions, or **(E-F)** total resection status. Statistical analysis was performed using Cox Proportional Hazards tests. Abbreviations: Gy = Gray, OS = Overall Survival, NS = not significant.

**Supplementary Table 2.** Patient Characteristics in patients with Medicare vs Private Insurance. Differences between the number of patients for each characteristic between the two cohorts were calculated. Age, BMI, distance from home to clinic, dose, fractions, and housing score were calculated as continuous variables, while the remaining variables were calculated as categorical variables. P values were calculated with two sample t-tests for continuous variables and Fisher’s exact tests for categorical variables. Abbreviations: Gy = Gray, BMI = Body Mass Index.

**Supplementary Figure 2.** Associations between insurance status, radiation treatments, age, and office visits. **(A)** Radiation dose, **(B)** number of radiation fractions, **(C)** age, and **(D)** total number of neurology, neurological surgery, or radiation oncology office visits in patients with Medicare or Private Insurance. **(D)** Relationship between total time elapsed from first to final office visits and total number of office visits for each patient, with calculated linear regression line and p values for slope differences. 95% CI for linear regression slope is 0.5985 to 0.7804 for patients with Medicare vs 0.5252 to 0.6455 for patients with Private Insurance. P values for were calculated with two sample t tests. Abbreviations: Gy = Gray.

**Supplementary Table 1.** Univariate analysis of impact of patient characteristics on OS and Cumulative Incidence in the full patient cohort.

|  | **OS** | | | **Cumulative Incidence** | | |
| --- | --- | --- | --- | --- | --- | --- |
| **Variable** | **Hazard Ratio** | **95% CI** | **p value** | **Hazard Ratio** | **95% CI** | **p value** |
| **Age** | 1.026 | 1.012 - 1.040 | 0.0002 | 1.011 | 0.997 - 1.026 | 0.1123 |
| **BMI** | 0.978 | 0.948 - 1.009 | 0.1597 | 1.006 | 0.973 - 1.040 | 0.7376 |
| **Distance from Clinic** | 1.000 | 0.998 - 1.002 | 0.7426 | 0.999 | 0.997 - 1.002 | 0.6583 |
| **Dose (Gy)** | 0.976 | 0.964 - 0.989 | 0.0002 | 0.984 | 0.968 - 0.999 | 0.0435 |
| **Fractions** | 0.958 | 0.939 - 0.978 | 3.5*10^-5^ | 0.967 | 0.943 - 0.991 | 0.0074 |
| **Housing Score** | 1.010 | 0.966 - 1.056 | 0.6768 | 1.008 | 0.958 - 1.060 | 0.7685 |
| **Gender (Male)** | 1.291 | 0.904 - 1.843 | 0.1598 | 1.311 | 0.874 - 1.966 | 0.1904 |
| **Race (Caucasian)** | 0.874 | 0.510 - 1.498 | 0.6247 | 0.928 | 0.495 - 1.740 | 0.8168 |
| **Marital Status (Married)** | 1.391 | 0.898 - 2.155 | 0.1397 | 1.406 | 0.851 - 2.324 | 0.1837 |
| **Employment (Employed)** | 0.757 | 0.531 - 1.080 | 0.1252 | 0.881 | 0.590 - 1.315 | 0.5344 |
| **Insurance (Private)** | 0.644 | 0.453 - 0.917 | 0.0146 | 0.843 | 0.552 - 1.286 | 0.4268 |
| **Smoking History** | 0.817 | 0.535 - 1.249 | 0.3508 | 0.714 | 0.432 - 1.180 | 0.1885 |
| **Anxiety Disorder** | 0.840 | 0.538 - 1.311 | 0.4423 | 0.899 | 0.546 - 1.480 | 0.6751 |
| **Depressive Disorder** | 0.809 | 0.571 - 1.144 | 0.2307 | 0.649 | 0.435 - 0.968 | 0.0340 |
| **Total Resection** | 0.602 | 0.403 - 0.900 | 0.0134 | 0.554 | 0.349 - 0.879 | 0.0122 |
| **Temozolomide Use** | 0.834 | 0.591 - 1.177 | 0.3014 | 0.796 | 0.537 - 1.180 | 0.2561 |
| **Dexamethasone Use** | 1.250 | 0.882 - 1.770 | 0.2092 | 1.129 | 0.758 - 1.682 | 0.5505 |
|  |  |  |  |  |  |  |

**Supplementary Figure 1.** Univariate impact of radiation dose, number of radiation fractions, and total resection status on OS and Cumulative Incidence in the full patient cohort.

**Supplementary Table 2.** Patient Characteristics in patients with Medicare vs Private Insurance

| **Characteristics** | **Medicare** | **Private Insurance** | **p value** |
| --- | --- | --- | --- |
| **Total Patients** | 63 | 106 |  |
| **Age** |  |  |  |
| **Median** | 71.2 | 54.9 | < 0.0001 |
| **Range** | 53.6 – 84.3 | 23.5 – 77.7 |  |
| **BMI** |  |  |  |
| **Median** | 27.3 | 28.3 | 0.9766 |
| **Range** | 18.8 – 45.2 | 18.8 – 48.8 |  |
| **Home Distance from Clinic (miles)** |  |  |  |
| **Median** | 25.7 | 26.7 | 0.4137 |
| **Range** | 1.8 – 637.0 | 1.8 – 395.0 |  |
| **Dose (Gy)** |  |  |  |
| **Median** | 40.0 | 55.0 | 0.2501 |
| **Range** | 18.7 – 75.0 | 16.0 – 75.0 |  |
| **Number of Fractions** |  |  |  |
| **Median** | 15 | 30 | 0.0053 |
| **Range** | 4 – 30 | 5 – 30 |  |
| **Housing Score** |  |  |  |
| **Median** | -0.81 | -0.51 | 0.1715 |
| **Range** | -5.61 – 8.74 | -5.61 – 19.60 |  |
| **Gender** |  |  |  |
| **Male** | 40 | 66 | 0.9999 |
| **Female** | 23 | 40 |  |
| **Race** |  |  |  |
| **Caucasian** | 61 | 91 | 0.0317 |
| **Non-Caucasian** | 2 | 15 |  |
| **Marriage Status** |  |  |  |
| **Married** | 52 | 81 | 0.4380 |
| **Not Married** | 11 | 25 |  |
| **Employment Status** |  |  |  |
| **Employed** | 7 | 63 | < 0.0001 |
| **Unemployed** | 56 | 43 |  |
| **Smoking History** |  |  |  |
| **Positive** | 18 | 20 | 0.1823 |
| **Negative** | 45 | 86 |  |
| **Anxiety Disorder History** |  |  |  |
| **Positive** | 9 | 21 | 0.4110 |
| **Negative** | 54 | 85 |  |
| **Depressive Disorder History** |  |  |  |
| **Positive** | 27 | 49 | 0.7497 |
| **Negative** | 36 | 57 |  |
| **Total Surgical Resection Status** |  |  |  |
| **Positive** | 49 | 80 | 0.8520 |
| **Negative** | 14 | 26 |  |
| **Concurrent Temozolomide Use** |  |  |  |
| **Positive** | 23 | 58 | 0.0261 |
| **Negative** | 40 | 48 |  |
| **Concurrent Dexamethasone Use** |  |  |  |
| **Positive** | 30 | 46 | 0.6334 |
| **Negative** | 33 | 60 |  |

**Supplementary Figure 2.** Associations between insurance status, radiation treatments, age, and office visits.
